# Firm size and economic concentration: An analysis from a lognormal expansion

**DOI:** 10.1371/journal.pone.0254487

**Published:** 2021-07-09

**Authors:** Lina M. Cortés, Juan M. Lozada, Javier Perote

**Affiliations:** 1 Universidad EAFIT, Medellín, Colombia; 2 University of Salamanca, Salamanca, Spain; Universidad de Castilla-La Mancha, SPAIN

## Abstract

This paper studies the distribution of the firm size for the Colombian economy showing evidence against the Gibrat’s law, which assumes a stable lognormal distribution. On the contrary, we propose a lognormal expansion that captures deviations from the lognormal distribution with additional terms that allow a better fit at the upper distribution tail, which is overestimated according to the lognormal distribution. As a consequence, concentration indexes should be addressed consistently with the lognormal expansion. Through a dynamic panel data approach, we also show that firm growth is persistent and highly dependent on firm characteristics, including size, age, and leverage −these results neglect Gibrat’s law for the Colombian case.

## 1. Introduction

The relationship between firm size (FS) and firm growth (FG) has been extensively studied since the early seminal study of Gibrat [1]. The so-called Gibrat’s law postulates that these two variables are not correlated, and the probability density function (PDF) of FS is stable and approximately lognormal. In an economy, many small businesses coexist with a few large companies, and Gibrat’s law is used as an explanation for the high bias in FS distribution [2–5]. Although this topic has been addressed in several studies, FS distribution is still an open question that arouses increasing interest among researchers and policymakers, since firm distribution is correlated with the degree of aggregate economic concentration and, consequently, is a cornerstone of antitrust policy [6–9].

However, in the literature, there is no consensus regarding the functional model that should be adopted to analyze FS distribution [10–12]. Although studies have found evidence that the lognormal distribution accurately fits to FS, favoring Gibrat’s law [13–15], other studies feature a poor performance of this distribution, especially in the higher quantiles [16–19]. In this line, some empirical studies have shown that FS distribution can be adjusted using a Pareto or Power-law distribution [7, 20–22], although this latter distribution presents the shortcoming of requiring the selection of a minimum threshold to assume that FS distribution is well defined [3, 23–27].

There is also a strand of literature that argues that the discrepancies on the data fits may be due to the fact that the distributions traditionally used to accommodate fat tails usually depend on very few parameters to determine the entire shape of the FS distribution, including the right tail of the distribution [18, 28–31]. This may result in density misspecification and misleading conclusions on economic policy recommendations, since FS dynamics is a determining factor of economic growth and stability. Note that, small changes in the way companies are distributed may have a significant macroeconomic effect, e.g., increased employment and income distribution [9, 32, 33].

Although several studies have investigated FS distribution, other research sought to understand what determines FG. In this framework, and taken Gibrat [1] as a reference, studies have mainly focused on analyzing the effects of FS distribution on FG [21]. However, the assumptions behind Gibrat’s law remain one of the most controversial and explored topics in the studies on industrial organization because the empirical evidence shows that in some industries or economies, FG depends on FS and/or company history [10, 33].

This study sheds some light on this topic with an empirical study based on Colombian firms. The motivation for choosing Colombia was that the empirical literature to date has focused on characterizing FS distribution and its growth determinants in other regions, including the United States, Europe, and Asia [2, 3, 9, 18, 23, 27, 34], but only a few papers [16, 35] have studied the growth of Latin American firms. Therefore, a study on this topic in a Latin American country represents a particularly relevant contribution to this literature, since companies in Latin America are characterized by a highly concentrated structure and less developed capital markets, even among emerging countries [36–38]. These conditions can generate potentially different results between the markets previously studied and the Latin American market.

In line with the above, this study has three primary objectives. The first is comparing the adjustment of FS distribution using the lognormal distribution [1] with the more flexible log-semi-nonparametric (log-SNP) distribution [31]. The log-SNP distribution, which generalizes the lognormal, is derived from a logarithmic transformation of SNP distributions based on Edgeworth and Gram-Charlier expansions. This transformation keeps the flexibility of the Gram-Charlier distributions’ parametric structure (i.e., the ability to asymptotically approximate the true density by adding more terms to the expansion) but constraining the domain to positive values. The log-SNP distribution has been applied in diverse fields in which the precision in the measurement of the distribution tails is crucial for accurately capturing the occurrence of extreme values. The studies by Kuhs [39], Blinnikov and Moessner [40], Mauleón and Perote [41], and Cortés et al. [42] have used this distribution in the areas of thermodynamics, astronomy, finance and scientometrics, respectively.

Secondly, firm distribution is closely associated with the level of economic concentration. In industrial economics studies, economic concentration has two dimensions: market concentration and aggregate economic concentration [43, 44]. On the one hand, market concentration is defined as the proportion of sales represented by a few large sellers concerning total production or sales in the market or industry. On the other hand, aggregate economic concentration corresponds to the degree to which a small number of large firms control the activity of an industry or economy (sales, profits, value-added). That is, the difference in the size of two firms (e.g., measured in terms of employment, sales, or assets) can provide a measure of the degree of “power” that one firm can exert over the other [6]. This study focuses on the second of the dimensions presented, and we use the sales variable as a proxy for FS.

In the context of aggregate economic concentration, the increase in inequality among firms results from changes in the size distribution of firms participating in the market, which is against Gibrat’s law. As a result, concentration tends to increase persistently in the long run. Fundamentally, concentration arises from the dispersion of FG rates and can be studied as the opposite of uniformity. The most commonly used techniques for studying aggregate economic concentration are the Lorenz curve and the Gini index [6, 17, 45, 46]. Starting from the definition proposed by Sen [47], we propose calculating the Gini index using the log-SNP distribution and comparing its performance with the lognormal distribution. From now on, we will refer to economic concentration to refer to aggregate concentration.

The third objective is to analyze the determinants of FG. To validate Gibrat’s law in an emerging Latin American market, we estimate the relationship between FG, FS, and other variables at the firm level. The empirical literature has found that other variables such as age or financial information can explain the growth of a firm. On the one hand, as firms age, they learn how to improve their productivity and acquire more information than that they use to have at the beginning of their operations [48]. As firms age, the firm’s products become better positioned, leading to increased reputation and customer loyalty, positively impacting FG [49]. Particularly, when analyzing the behavior of Colombian firms, this variable is relevant since the proportion of firms with more than ten years within the large segment is around 70%, while for the small segment, it is 21% [50].

On the other hand, financial factors can increase or restrict a firm’s growth. More profitable firms have greater resources to execute their investments so they can grow more. As suggested by the pecking order theory, firms turn first to internal resources, and then to external resources. In addition, the ability of firms to access credit can provide information about their future growth [51]. In the Colombian case, firms have a high ownership concentration and their access to the capital market is limited, so their main source of financing is credit. However, small and medium-sized enterprises (SMEs) have historically had less access to credit because financial institutions consider them riskier [52]. This fact is relevant when analyzing the evolution of the FG since credit restrictions have a negative relationship with the growth of firms [53]. Considering how relevant these facts can be when analyzing Colombian firms, we decided to use as control variables profitability (ROE), leverage (debt-to-total assets ratio), and age. We calculate FG as the first difference of the natural logarithm of sales. The dynamic panel methodology proposed by Arellano and Bond [54] and Blundell and Bond [55] was used to control for the endogeneity and unobservable heterogeneity associated with this type of models.

Our results evidenced that Gibrat’s law did not apply to the Colombian economy during the period of study. Compared with the lognormal distribution, the log-SNP distribution provided a better fit when modeling FS distribution. Moreover, the log-SNP distribution allowed a better adjustment in the upper quantiles without imposing a minimum threshold, which allowed us to obtain a better quantification of the Gini index. This is relevant because knowing the characteristics of larger companies and having a larger share of the market is essential to analyze the entire economy. In addition to variables such as growth rate and the correlation between FG and FS, we find that variables linked to size, age, and leverage are fundamental determinants of FG.

The rest of the paper is structured as follows. Section 2 contains definitions about FS distribution and a description of the log-SNP distribution. Section 3 defines the economic concentration and approaches to its quantification using the log-SNP distribution. Section 4 reviews the relevant literature on the determinants of FG and presents the hypotheses to be analyzed. Section 5 reports the collected data and descriptive statistics on the evaluated variables. Section 6 describes the results of the comparison of the performance of lognormal and log-SNP distributions and discusses their compliance with Gibrat’s law. The last section summarizes the conclusions.

## 2. Firm size distribution

Gibrat [1] proposed that FS distribution is adequately estimated using a lognormal distribution because FG tends to be multiplicative and independent of its size at a certain point in time. Formally, let *z*_*t*_∈ℝ^+^ be a random variable (with finite variance) that represents FS at a time *t*, and let *γ*_*t*_ denote its corresponding growth rate, i.e. *z*_*t*_ = *δ*_*t*_*z*_*t*−1_, where *δ*_*t*_ = 1+*γ*_*t*_. It follows that

*z*_*t*_ = *δ*_*t*_*z*_*t*−1_ = *δ*_*t*_*δ*_*t*−1_*z*_*t*−2_ = ⋯ = *δ*_*t*_*δ*_*t*−1_⋯*δ*_1_*z*_0_, and in logarithmic form

ln(*z*_*t*_) = ln(*δ*_*t*_)+ln(*δ*_*t*−1_)+⋯+ln(*δ*_1_)+ln(*z*_0_).

Assuming that the terms ln(*δ*_*j*_), with *j* = 1,…,*t* are independent and identically distributed, and applying the central limit theorem, it can be concluded that ln(*z*_*t*_)∈ℝ approximately follows a normal distribution and thus *z*_*t*_ is lognormal distributed [10, 26].

Therefore, a strand of empirical literature has been devoted to the evaluation of the performance of lognormal distribution using cross-sectional data on FS [14–16] finding that this distribution seems to either underestimate or overestimate the theoretically expected values in different ranges of the upper quantiles of FS distribution. Consequently, Cortés et al. [31] proposed modeling FS using the log-SNP distribution, as described below.

Analyzing FS distribution based on cross-sectional data, let *z*_*i*_ be the variable that measures FS at a specific time; then, it is said to be log-SNP distributed if its PDF can be expressed as

$$h(z_{i};\;\mu ,\sigma^{2},d)=\left(\frac{1}{z_{i}\sigma \sqrt{2\pi }}e^{-\frac{{\left(\ln\left(z_{i}\right)-\mu \right)}^{2}}{2\sigma^{2}}}\right)\left[1+\sum_{s=1}^{n}d_{s}H_{s}(\frac{\ln\left(z_{i}\right)-\mu }{\sigma })\right],\;z_{i}\in R^{+},$$ (1)

where *μ*∈ℝ and *σ*^2^∈ℝ^+^ represent the location and scale, respectively, ***d*** = (*d*_1_,…,*d*_*n*_)′∈ℝ^*n*^ are shape parameters and *H*_*s*_(*x*_*i*_) is the Hermite polynomial (HP) of order *s*, which is defined as the *s*-th order derivative of $\phi (x_{i})\frac{1}{\sqrt{2\pi }}e^{-\frac{1}{2}x_{i}^{2}}$,

$$\frac{d^{s}\phi (x_{i})}{{dx_{i}}^{s}}={\left(–1\right)}^{s}H_{s}(x_{i})\phi (x_{i}),$$ (2)

e.g., the first four HPs are *H*_0_(*x*_*i*_) = 1, *H*_1_(*x*_*i*_) = *x*_*i*_, $H_{2}(x_{i})=x_{i}^{2}–1,\;H_{3}(x_{i})=x_{i}^{3}–3x_{i}$, and $H_{4}(x_{i})=x_{i}^{4}–6x_{i}^{2}+3$.

It is noteworthy that the lognormal distribution is a particular case when ***d*** = 0. Consequently, as well as the lognormal corresponds to an exponential transformation of the normal, the log-SNP is the exponential transformation of a variable with SNP distribution (also known as Gram-Charlier Type A). That is, *z*_*i*_ = exp (*x*_*i*_) if *x*_*i*_ has an SNP distribution, i.e. its PDF is of the type:

$$f(x_{i};d)=[1+\sum_{s=1}^{n}d_{s}H_{s}(x_{i})]\phi (x_{i}),\;x_{i}\in \mathbb{R},$$ (3)

Furthermore, the HPs form an orthonormal basis and therefore satisfy the following orthogonality property,

$$\int_{-\infty }^{\infty }H_{s}(x_{i})H_{j}(x_{i})\phi (x_{i})dx_{i}=0\;\forall s\neq j,$$ (4)

which is the ground for interesting results as the fact that the expansion integrates to one or that the even (odd) *k-*order moment only depends on *d*_*s*_, for *s*≤*k* and *s* being even (odd) parameters, e.g. *d*_1_ and *d*_2_ account for mean and variance, *d*_3_ and *d*_4_ incorporate bias and excess kurtosis (provided that *d*_1_ = *d*_2_ = 0), respectively, and the remaining parameters represent higher-order moments. It is clear that the parameter flexibility of the SNP density represents a major advantage compared to other traditional densities that depend on a limited number of parameters. However, it is noteworthy that for finite expansions non-negativity is not guaranteed for all ***d***∈ℝ^*n*^, and thus different studies have considered positive transformations [56] or positivity restrictions [57]. Our empirical study does not constrain the maximum likelihood optimization of the Gram-Charlier expansion but implements motorized estimation to ensure the converge of the algorithms to values that guarantee a well-defined PDF.

## 3. Firm size and economic concentration

Firm distribution is closely related to the level of economic concentration [6, 17]. In this respect, the Gini index provides an average measure of dominance within a group of companies, and thus this measure can be used to compare the evolution in FS distribution with the evolution of economic concentration [29, 45, 46]. Since the Gini index is based on the Lorenz curve, several models of that curve have been developed in the economic literature [29, 58–60]. However, according to Sen [47], in an empirical sample {*z*_1_,…,*z*_*n*_}, the Gini index can be estimated using the discrete equation

$$\hat{Gini}=\frac{1}{n}[n+1-2\frac{\sum_{i=1}^{n}(n+1-i)z_{i}^{*}}{\sum_{i=1}^{n}z_{i}^{*}}],$$ (5)

where $z_{i}^{*}$ is the order statistic.

According to Gibrat’s law, the PDF of an empirical sample can be fitted using the lognormal distribution, which assumes the cumulative distribution function (CDF)

$$F(z_{i};\;\mu ,\sigma^{2})=\Phi \left(\frac{\ln\left(z_{i}\right)-\mu }{\sigma }\right)=\int_{0}^{z_{i}}\frac{1}{\sigma q_{i}\sqrt{2\pi }}e^{-\frac{{\left(\ln\left(q_{i}\right)-\mu \right)}^{2}}{2\sigma^{2}}}dq_{i}.$$ (6)

However, some authors proposed using non-parametric or semi-nonparametric distributions to fit the empirical sample and estimate the Gini index described in Eq (5) [61, 62]. Considering that many factors may affect the degree of economic concentration, it can be difficult to summarize the characterization of FS distribution using a few parameters. For instance, when FS distribution is widely dispersed around the mean, and larger companies are relatively large, it may be more challenging to determine extreme values with traditional parametric distributions [6].

The lognormal is nested in the log-SNP, the latter being a natural alternative for testing the need for additional parameters to capture the density assessment at the upper quantiles. Even more, the of the CDF of the log-SNP can be directly obtained − see Eq (7) − distribution can be obtained and used for computing the probabilities and quantiles of this distribution.


$$\begin{array}{l} F(z_{i};\;\mu ,\sigma^{2},d)=\int_{0}^{z_{i}}(\frac{1}{\sigma q_{i}\sqrt{2\pi }}e^{-\frac{{\left(\ln\left(q_{i}\right)-\mu \right)}^{2}}{2\sigma^{2}}})[1+\sum_{s=1}^{n}d_{s}H_{s}(\frac{\ln\left(z_{i}\right)-\mu }{\sigma })]dq_{i},\\ \;=\Phi \left(\frac{\ln\left(z_{i}\right)-\mu }{\sigma }\right)-\phi \left(\frac{\ln\left(z_{i}\right)-\mu }{\sigma }\right)\sum_{s=1}^{n}d_{s}H_{s-1}(\frac{\ln\left(z_{i}\right)-\mu }{\sigma }). \end{array}$$ (7)

This large number of parameters does not result in higher computational difficulty and can be obtained by maximum likelihood (ML), whose log-likelihood (logL) function is given by:

$$logL(z_{i};\;\mu ,\sigma^{2},d)=-\frac{1}{2}\log\left(2\pi \sigma^{2}z_{i}^{2}\right)-\frac{1}{2}{\left(\frac{\log\left(z_{i}\right)-\mu }{\sigma }\right)}^{2}+\log\left[1+\sum_{s=1}^{n}d_{s}H_{s}(\frac{\log\left(z_{i}\right)-\mu }{\sigma })\right].$$ (8)

A straightforward procedure for the selection of the expansion order consists of starting with the lognormal logL and recursively adding *d*_*s*_ parameters according to Akaike’s Information Criteria (AIC) and likelihood ratio (LR) tests. The quantiles of the log-SNP distribution are directly retrieved from the Eq (8).

## 4. Firm size and its determinants

This section reviews the relevant literature on the determinants of FG and discuss some conjectures underlying the role of firm characteristics on explaining FG using Gibrat’s law. As a by-product, we establish a model to empirically evaluate Gibrat’s law compliance.

### 4.1. Theoretical background and hypothesis formulation

On the grounds of Gibrat’s [1] seminal paper, several authors have investigated the relationship between FS and FG [5, 7, 63]. According to Gibrat’s law, FG rates do not depend on the FS and/or company history. That is, the distribution of FG rates in an economy is identical for all companies, regardless of their current size and/or previous growth history.

However, some studies have questioned the validity of Gibrat’s law [10, 34, 64–66]. Among them, there are several opinions on the determinants of FG [67] because the growth patterns may depend on different factors, which were corroborated in previous theoretical and empirical studies. For instance, in addition to FS, other variables may affect firm dynamics and evolution [34, 53, 68, 69].

Gibrat’s law can be tested using three different approaches: (i) considering all the companies within an industry or a specific economy and time interval, including the companies that did not survive; (ii) considering only surviving companies; (iii) considering companies large enough to reach the minimum efficiency scale [70]. However, the available studies have focused mainly on the second approach. In the Colombian context, few studies have addressed the determinants of FG with information at the firm level. In part, this problem is due to the difficulty of having a relatively comprehensive database in both dimensions: temporal and representative of the different sizes of firms. In many cases, the available information is not of the best quality, and there is no clarity regarding the entry and exit of companies. Based on the quality of the Colombian data, this study is carried out in the second approach.

In this respect, it is necessary to correct heteroscedasticity and serial correlation when analyzing the determinants of FG in a sample of surviving companies because, if the study is based only on surviving companies, it is very likely that sample selection is strongly correlated with the same variables that may affect FG [11, 19, 34, 53, 68]. To confirm the validity of Gibrat’s law and the impact of other variables on company growth, several studies have focused on dynamic econometric models [8, 34, 51, 53, 71, 72]. In the present research, in addition to evaluating the relationship between company growth and size, other determinants were considered, including firm age, leverage, and profitability.

When analyzing FG, Gibrat’s law assumes the absence of autocorrelation in errors or non-persistence of the growth rate. However, previous studies using dynamic econometric models provided evidence of growth rates persistence. However, the magnitude and direction of this effect are not entirely clear [33]. For instance, some studies found that the growth rate in a specific period was positively correlated with its first lag in growth [34, 53, 73–75]. Other studies reported that negative persistence values indicated that firms with slow growth rates in the past will tend to grow less in the future [5, 51, 63, 64, 72, 75]. This leads to the following testable hypothesis, which is not supported by the Gibrat’s law:

*Hypothesis 1*. *FG is expected to be persistent in time*.

On the other hand, Gibrat’s law postulates the lack of correlation between FG and FS. However, empirical studies point to the opposite result [34, 76, 77]. Firm size can be measured using different parameters, including sales, assets, employees, and benefits, among others [9, 18, 67, 78]. The number of employees, assets, and sales are the most frequently used. However, each of these measures has advantages and disadvantages. The number of employees is a discrete variable that may not reflect the increase in employee productivity [79]. The level of assets, in contrast to the number of employees and level of sales, can assume negative values [76]. Therefore, previous studies suggest that the level of sales may better represent FS [26, 31]. Based on the different measures previously presented, authors have found that firm growth inversely relates to firm size. This negative relationship implies that smaller firms grow faster than larger ones, seeking to reach a minimum efficient size [8, 68, 71]. Thus, we conjecture the following hypothesis:

*Hypothesis 2*. *FS is negatively related to FG*.

In addition, empirical studies found that FG might be affected by age [53, 66, 77]. In this respect, Evans [64], Reid and Xu [80] and Barba Navaretti et al. [8] found a negative relationship between firm age and growth, that is, young companies developed faster than their older counterparts. In contrast, Das [49] and Shanmugam and Bhaduri [81] show a positive relationship between FG and firm age. According to Das [49], the positive effect may be because over the years, consumers become more aware of the existence of a product or service, which increases their consumption and thus result in greater growth in the firm. Furthermore, the firm’s reputation can improve with age and this can be reflected in a positive impact. Also, some authors have evaluated the presence of non-linear relationships. Park et al. [82] found a concave relationship between FG and firm age, suggesting that FG decreased more rapidly as companies aged. Accordingly, we posit two testable hypotheses:

*Hypothesis 3a*. *Firm age linearly affects FG*.
*Hypothesis 3b*. *Firm age nonlinearly (quadratically) affects FG*.

Studies on FG used leverage as a control variable [8, 51]. Theoretically, leverage generates benefits [83] and costs (e.g., financial difficulties and agency costs; Jensen [84]) which may have variable effects on growth. The studies by Jang and Park [85] and Canarella and Miller [34] found a negative relationship between the level of leverage and FG rate. This result is because companies lose financial flexibility as they become more indebted, which may lead to the rejection of projects with a positive net present value in inefficient markets, and consequently less growth. In contrast, Huynh and Petrunia [51] and Barba Navaretti et al. [8] found a positive association between the level of leverage and FG. The reason is because debt is a mechanism of control used by shareholders over managers. If a company has debts, the manager should be more efficient and pay debts by avoiding waste and poor investments. In addition, a positive relationship can be explained by companies’ desire to avoid raising capital and the consequent loss of control [37]. This leads to the following testable hypothesis regarding financial leverage impact on FG:

*Hypothesis 4*. *Financial leverage causes a positive effect on FG*.

Finally, and according to the pecking order theory, companies initially prefer to finance investment projects by reinvesting profits because the asymmetry of market information can make other sources of financing more expensive [86]. In this respect, it is expected that companies with higher profitability can make investments with lower costs and therefore, grow more. Jang and Park [85] and Canarella and Miller [34] found empirical evidence that supports a positive link between profitability and FG. In contrast, Heshmati [87] and Liñares-Zegarra and Wilson [75] found that there was no significant relationship between profitability and FG. This leads to the following conjecture on the relation between firm profitability and growth:

*Hypothesis 5*. *Profitability (ROE) generates a positive effect on FG*.

### 4.2. Econometric modeling

In order to test the relation between FG and FS implicit in the Gibrat’s Law, as well as the impact of other characteristics related to the Colombian firms, we propose the following model:

$${Growth}_{it}=\alpha_{i}+\beta {Growth}_{i,t-1}+\gamma \;\log\left({Sales}_{t-1}\right)+\theta_{1}log{Age}_{it}+\theta_{2}{\left[log{Age}_{it}\right]}^{2}+\varphi {Leverage}_{i,t-1}+\omega {ROE}_{i,t-1}+\varepsilon_{it},$$ (9)

where *Growth*_*it*_ is FG calculated as the first logarithmic difference of sales; *Growth*_*i*,*t*−1_ is the first lag of FG; log(*Sales*_*t*−1_) is a proxy of FS measured as the natural logarithm of sales, all for a specific firm *i* and time *t*; *logAge*_*it*_ is the logarithm of the age of the company since its foundation, which is considered in both level and quadratic form; *Leverage*_*i*,*t*−1_ is the first lag of leverage calculated as the sum of the long-term debt and short-term debt divided by the total assets; and *ROE*_*t*−1_ is the first lag of profits, calculated as the net profit divided by common equity. Furthermore, *α*_*i*_ and *ε*_*it*_ correspond to the unobserved fixed effect of the company and the error term (which holds the standard assumptions of panel data models), respectively.

Given the dynamic panel data nature of Eq (9) estimation was performed by the generalized method of moments (GMM) estimator developed by Arellano and Bond [54]. This estimator uses the lagged levels as valid instruments of the of the differenced variables and induces first order, but not second-order, correlation in the estimated first-differenced model. However, the GMM difference estimator may produce weak instruments if the parameter of interest is close to one, which results in biased and inconsistent finite sample properties. Blundell and Blond [55] proposed using the system GMM estimator to address the problem. The system estimator uses the lagged differences in endogenous variables, in addition to the variables used in the original estimator. Consequently, system GMM presents a superior performance in finite samples than the difference estimator.

## 5. Data description and statistics

This study analyzes a sample of Colombian companies from 2002 to 2015. The primary sources of information were reports of financial statements, annexes, and basic information that companies send annually to the Superintendence of Companies of Colombia [88]. This source reports valuable information at the firm level but also has several limitations. Despite the legal provisions that oblige companies in Colombia to present financial statements annually, data from some companies in the database of the Superintendence are available for specific time periods but not for others, which limits the control for inclusion and exclusion criteria. Since the EMIS Benchmark was used to verify data consistency, the sample restricted to 1,772 surviving companies from all economic sectors. However, there were no restrictions on the minimum level of net sales. The sample included large and small companies, in contrast to other studies on FG, which focused on either large or small companies.

Some descriptive statistics for the entire sample period (2002–2015), particularly the first four moments, for each variable are summarized in Table 1. The third and fourth central moments provided useful information about firm distribution shape, and the means and standard deviations are also provided. The variable sales, which in this case was related to FS, featured positive asymmetry, with a very high number of small businesses. Positive kurtosis also indicated that the upper quantile of the distribution was larger than that of a lognormal distribution. On average, companies were at a mature age. However, there was high variability in growth rate. On average, Colombian companies had a high level of leverage and high variability in profits.

**Table 1:** Descriptive statistics.

Variable	n	Mean	Sd	Skew	Kurtosis
Growth	24,808	1.61%	48.85%	0.78	46.00
Sales	24,808	22,447	68,140	20.08	689.07
Age	24,808	30.04	10.30	0.68	4.21
Leverage	24,808	40.98%	27.67%	2.16	26.01
ROE	24,808	2.52%	654.72%	-116.96	17896.12

Note: The sample is composed of 1,772 Colombian companies in all sectors of the economy. The data is collected over a 14-year period from 2002 to 2015. Growth is the firm’s growth, calculated as the first difference of the natural logarithm of sales. Sales is the value of net sales in Colombian peso (COP) deflated by Colombian Consumer Price Index (CPI) using 2008 price base. Age corresponds to the age measured in years. Leverage is calculated as the sum of long-term debt and short-term debt divided by total assets. ROE is computed as net income divided by common equity. n = number of observations, Mean = mean value of the variable, Sd = standard deviation, Skew and Kurtosis correspond to the coefficient of asymmetry and excess kurtosis, respectively.

**Fig 1:**
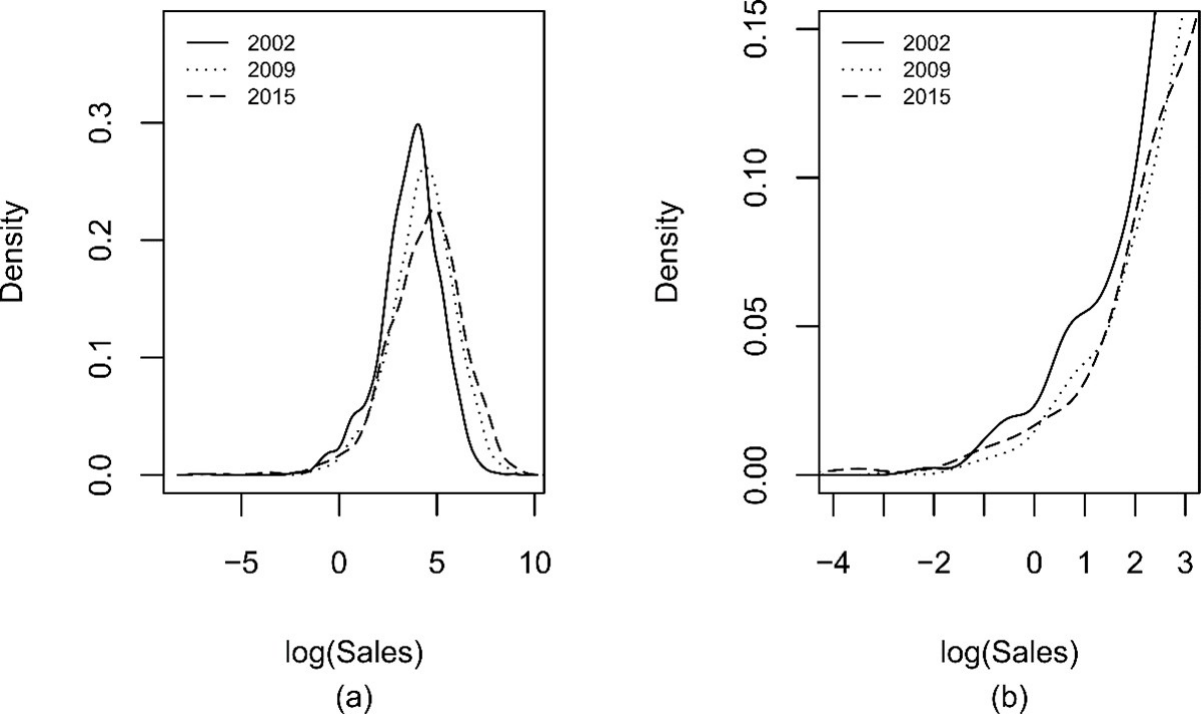
Empirical density of the logarithm of sales. The figure shows the density of the logarithm of the sales variable (log (Sales)) resulting from a smoothing of the corresponding histogram. The panel (a) represents the total of the domain and (b) a detail of the left tails, where the smallest firms in terms of sales are located. Sales were divided by the factor 10^8^.

The graph of the density of the logarithm of sales resulting from smoothing of the corresponding histogram is presented in Fig 1. The years 2002, 2009, and 2015 were selected at random to visualize the densities better. The picture shows density dynamics, illustrating its deviations from the lognormal distribution over time. Long-term FS distribution becomes more dispersed near the mean, more biased toward small firms, and larger in the higher quantiles. The empirical evidence from Kernel density estimation indicated that the shape of FS distribution was different from that of the lognormal distribution (Fig 1A). Furthermore, the tail (Fig 1B) featured multimodality or jumps, as observed by Reichstein and Jensen [18], Marsili [89], Bottazzi et al. [90], and Cortés et al. [31].

## 6. Results and discussion

This section presents and discusses the results of FS distribution, economic concentration, and the determinants of FG.

### 6.1. Modeling FS distribution

Table 2 reports the ML estimates obtained from Eq (13) for lognormal distribution (Panel A) and log-SNP distribution (Panel B). The results indicate that both models adequately determined the mean and standard deviation of the sample of selected companies. These statistics are represented by the location (μ) and scale (σ) parameters, respectively. The p-values indicate that these parameters are highly significant for both distributions. However, the parameters *d*_*s*_ were also highly significant for most of the evaluated years in the log-SNP distribution (Panel B).

**Table 2:** Estimates of firm size distribution using a lognormal and log-semi-nonparametric distribution.

Year	Panel A lognormal					Panel B log-SNP									Panel C LR
	μ	σ	logL	AIC	KS test	μ	σ	d_1_	d_2_	d_3_	d_4_	logL	AIC	KS test	
2002	3.6133	1.5879	-9,736.50	19,476.99	(0.0045)	1.4768	1.5608	1.3689	0.9545	0.3476	0.0690	-9,668.80	19,349.60	(0.9184)	135.39
	(< .0001)	(< .0001)			Rejected	(< .0001)	(< .0001)	(< .0001)	(< .0001)	(< .0001)	(< .0001)			Not rejected*	(< .0001)
2003	3.7475	1.5881	-9,974.50	19,952.99	(0.0253)	1.7825	1.5169	1.2954	0.8870	0.3136	0.0628	-9,922.35	19,856.71	(0.4552)	104.28
	(< .0001)	(< .0001)			Not rejected	(< .0001)	(< .0001)	(< .0001)	(< .0001)	(< .0001)	(< .0001)			Not rejected*	(< .0001)
2004	3.8629	1.5986	-10,190.67	20,385.34	(0.0410)	1.9147	1.5299	1.2734	0.8567	0.2926	0.0561	-10,138.89	20,289.78	(0.8099)	103.57
	(< .0001)	(< .0001)			Not rejected	(< .0001)	(< .0001)	(< .0001)	(< .0001)	(< .0001)	(< .0001)			Not rejected*	(< .0001)
2005	3.9511	1.5829	-10,329.62	20,663.24	(0.0308)	2.3932	1.4447	1.0784	0.6816	0.2075	0.0373	-10,292.80	20,597.61	(0.6455)	73.63
	(< .0001)	(< .0001)			Not rejected	(< .0001)	(< .0001)	(< .0001)	(< .0001)	(0.004)	(0.0401)			Not rejected*	(< .0001)
2006	4.0923	1.6524	-10,655.87	21,315.74	(0.383)	2.0231	1.5973	1.2954	0.8741	0.2963	0.0627	-10,599.79	21,211.59	(0.8099)	112.15
	(< .0001)	(< .0001)			Not rejected	(< .0001)	(< .0001)	(< .0001)	(< .0001)	(< .0001)	(< .0001)			Not rejected*	(< .0001)
2007	4.2018	1.6793	-10,878.49	21,760.98	(0.0169)	2.1939	1.5899	1.2629	0.8553	0.2939	0.0627	-10,826.84	21,665.68	(0.8346)	103.31
	(< .0001)	(< .0001)			Not rejected	(< .0001)	(< .0001)	(< .0001)	(< .0001)	(< .0001)	(< .0001)			Not rejected*	(< .0001)
2008	4.2697	1.6560	-10,974.08	21,952.16	(0.0836)	4.4345	1.7820	-0.0925	-0.0640	-0.0435	0.0235	-10,949.07	21,910.15	(0.6455)	50.01
	(< .0001)	(< .0001)			Not rejected	(< .0001)	(< .0001)	(0.4161)	(0.1352)	(0.0555)	(0.0006)			Not rejected*	(< .0001)
2009	4.2478	1.6638	-10,943.68	21,891.35	(0.2445)	2.0663	1.6910	1.2901	0.8162	0.2760	0.0494	-10,915.71	21,843.42	(0.9001)	55.93
	(< .0001)	(< .0001)			Not rejected	(< .0001)	(< .0001)	(< .0001)	(< .0001)	(< .0001)	(< .0001)			Not rejected*	(< .0001)
2010	4.2745	1.6774	-11,005.39	22,014.78	(0.2797)	3.0176	1.5149	0.8297	0.4573	0.1105	0.0295	-10,987.51	21,987.02	(0.6455)	35.77
	(< .0001)	(< .0001)			Not rejected	(< .0001)	(< .0001)	(< .0001)	(< .0001)	(0.0243)	(0.0546)			Not rejected*	(< .0001)
2011	4.3511	1.7271	-11,192.81	22,389.61	(0.0056)	1.9534	1.8060	1.3277	0.8386	0.2802	0.0533	-11,163.85	22,339.69	(0.8346)	57.92
	(< .0001)	(< .0001)			Rejected	(< .0001)	(< .0001)	(< .0001)	(< .0001)	(< .0001)	(< .0001)			Not rejected*	(< .0001)
2012	4.3619	1.7426	-11,227.72	22,459.44	(0.2445)	2.3906	1.7170	1.1481	0.6740	0.2068	0.0345	-11,205.56	22,423.11	(0.6455)	44.33
	(< .0001)	(< .0001)			Not rejected	(< .0001)	(< .0001)	(< .0001)	(< .0001)	(0.0019)	(0.0145)			Not rejected*	(< .0001)
2014	4.3607	1.8548	-11,336.24	22,676.47	(0.383)	1.8202	1.9081	1.3314	0.8588	0.2830	0.0559	-11,293.32	22,598.63	(0.9001)	85.84
	(< .0001)	(< .0001)			Not rejected	(< .0001)	(< .0001)	(< .0001)	(< .0001)	(< .0001)	(< .0001)			Not rejected*	(< .0001)
2015	4.3796	1.9404	-11,449.71	22,903.42	(0.5071)	1.5302	2.0167	1.4129	0.9611	0.3281	0.0704	-11,389.43	22,790.86	(0.7299)	120.55
	(< .0001)	(< .0001)			Not rejected	(< .0001)	(< .0001)	(< .0001)	(< .0001)	(< .0001)	(< .0001)			Not rejected*	(< .0001)

Note: This table reports ML estimates for a sample of 1,772 Colombian firms. Sales were divided by the factor 10^8^. Panels A, B and C show the estimated parameters for the lognormal distribution, log-SNP distribution and the likelihood ratio for testing both specifications, respectively. *μ* and *σ* are location and scale parameters (respectively) and *d*_*s*_ the shape parameters. logL = log-likelihood, AIC = Akaike Information Criterion, LR = likelihood ratio, KS test = Kolmogorov-Smirnov test. Not rejected* indicates a better fit in the KS test. P-values in parentheses.

The analysis of the AIC statistic, which penalizes the inclusion of additional parameters in the two distributions, indicates that this criterion is consistently lower in the log-SNP distribution, suggesting that the model for this distribution provides a better performance. According to the Kolmogorov-Smirnov (KS) test, neither the lognormal nor the log-SNP can be rejected as the data generating process at a 1% significance level and for most of the years. However, the LR statistic for the difference between the log-SNP and lognormal distribution, shown in panel C, presents strong evidence in favor the log-SNP specification. The results of this test confirm the fact that the incorporation of the parameters *d*_*s*_ is significant and leads to the log-SNP model outperformance. This means that FS distribution presents significant asymmetries (captured by parameter *d*_3_) and non-monotonic thick tails (captured by parameter *d*_4_), due to the presence of extreme values, which definitely cannot be represented by the lognormal distribution.

**Fig 2:**
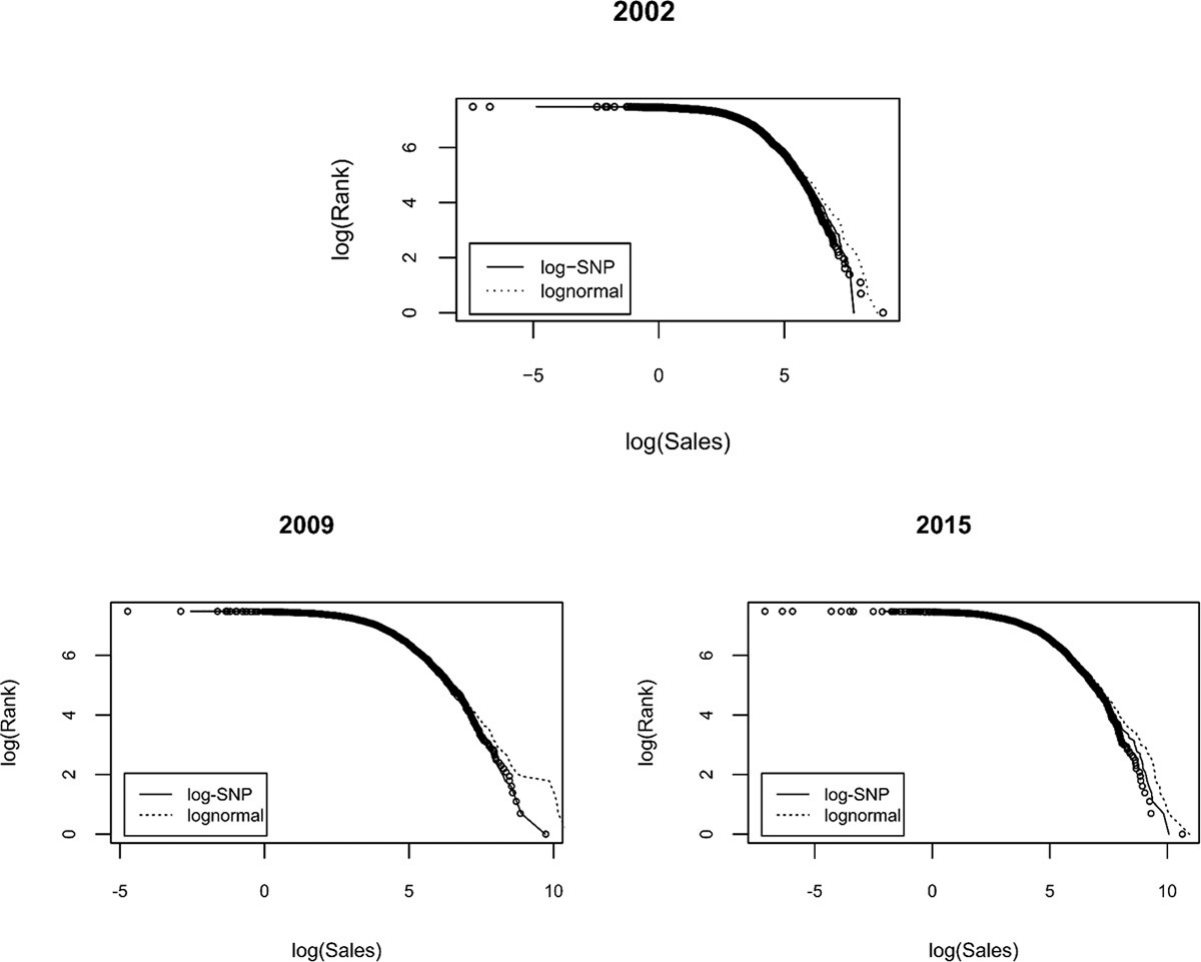
Logarithm of firm size vs. logarithm of sales. The figure compares the empirical values (hollow points) and the estimated values under a lognormal specification (dashed line) and log-SNP (solid line). The axes are in logarithmic scale and correspond to the relationship between Rank and Sales for a sample of 1,772 Colombian firms. Sales were divided by the factor 10^8^.

The relationship between rank and sales (in logarithmic scale) for the years 2002, 2009, and 2015 is shown in Fig 2. The comparison of empirical values (hollow points) and those estimated using a lognormal distribution (dashed line) and log-SNP distribution (solid line) reveals that the log-SNP captured more adequately the empirical distribution. The parameter *σ*^2^ captures the full shape of the lognormal distribution, which may induce that the expected values in the far end of the distribution tails tend to be systematically overestimated, as previously reported for other regions [2, 3, 19, 31]. For the log-SNP distribution, the parameter *σ*^2^ concentrates on explaining the variability around the mean (extreme values and skewness being accounted by *d*_4_ and *d*_3_, respectively). Even more, the variance of the SNP is *σ*^2^(1+2*d*_2_); thus, dispersion around the mean depends on both parameters. In the results reported in Table 2, negative values of *d*_1_ capture the decreasing in conditional mean of FS provoked by the 2008 recession. The negative value of *d*_2_ implies a reduction in the FS distribution variance (i.e., variability around the mean), which is compensated by an increase in negative skewness (*d*_3_<0) and kurtosis (*d*_4_>0). To obtain the quantiles of the distribution, we generated the random variable from the Inverse Transform Method, which computationally involves the use of the inverse of the CDF [91]. In the case of the log-SNP distribution, we use the inverse function from de CDF presented in Eq (7), and the lognormal is a particular case where ***d*** = **0**.

As an additional robustness test and to compare the performance of the lognormal and log-SNP distributions against the Pareto distribution, we conducted a further analysis based on the Generalized Pareto Distribution (GPD). We calculate the upper quantile of the sales distribution at a confidence level of 10%, 5%, and 1%. We use the parameters obtained from the estimation with the 5% and 10% threshold to calculate the GDP quantiles. When comparing the lognormal and GDP distributions, the results are very similar in the upper quantiles (i.e. for capturing extreme values), and the log-SNP presents a superior performance (results on these analyses are available upon request).

### 6.2. Analysis of economic concentration

Under Gibrat’s law, the Gini index presented in Eq (5) should be calculated using the values predicted theoretically by the CDF of the lognormal distribution described in Eq (6). However, there is still controversy regarding the distribution function that best represents the upper quantiles, especially for extreme values [60]. In this respect, Hart and Prais [6] reported that, in the case of lognormal distribution, changes in the parameter of the scale σ were positively correlated with changes in the level of economic concentration. However, these changes may be the result of different factors that may affect the degree of competition and, in that case, it may be difficult to summarize the changes using a single parameter.

In this respect, the present study used the log-SNP distribution to analyze the economic concentration, measured from sales in the sample of the selected companies. We expect that the flexible parametric structure of the log-SNP distribution may allow a better adjustment of the predicted values in the presence of heavy tails. The sales, in millions of Colombian pesos, obtained empirically for the sample of 1,772 Colombian companies versus the values expected theoretically using a lognormal distribution and log-SNP distribution are shown in Table 3. The analysis of the trend of the upper quantile of the distribution of sales at a confidence level of 10%, 5%, and 1% indicated the errors in the estimation of FS distribution using a lognormal distribution, possibly leading to an inadequate measurement of the level of economic concentration.

**Table 3:** Sales obtained empirically versus values expected theoretically using a lognormal distribution and log-semi-nonparametric distribution.

Year	Observed sales value (millions, COP pesos)			Expected sales value (millions, COP pesos)					
				Lognormal			Log-SNP		
	10%	5%	1%	10%	5%	1.0%	10%	5%	1%
2002	23,451.86	38,227.88	87,209.13	28,378.75	50,526.95	149,099.32	24,018.42	39,525.33	101,613.90
2003	28,717.54	46,491.08	99,324.82	32,463.40	57,804.70	170,603.97	27,501.74	44,909.17	113,434.40
2004	31,337.34	50,769.05	119,950.90	36,928.78	66,007.68	196,216.30	31,326.16	51,456.53	131,399.60
2005	35,026.29	57,931.51	126,561.90	39,533.83	70,262.34	206,642.20	34,191.24	55,511.25	137,742.60
2006	42,617.09	72,973.43	170,576.80	49,767.04	90,710.48	279,715.40	42,148.36	70,741.10	188,147.10
2007	50,030.84	86,731.06	210,178.50	57,472.96	105,785.12	332,236.20	48,449.59	81,331.37	216,017.80
2008	54,082.71	95,918.84	212,051.90	59,701.06	108,958.68	336,803.50	54,025.03	91,018.41	236,091.90
2009	53,869.23	93,628.22	242,631.30	58,999.08	107,984.97	335,585.60	52,740.64	91,338.40	257,938.90
2010	57,660.52	107,134.72	247,459.70	61,660.73	113,416.07	355,748.80	56,445.67	96,872.13	263,597.20
2011	64,912.65	117,687.80	271,429.20	70,941.61	132,860.90	431,077.00	64,208.30	115,175.00	348,034.50
2012	67,081.24	123,506.30	308,754.10	73,150.65	137,773.90	451,777.60	65,657.94	116,254.40	340,606.30
2013	68,343.02	128,521.50	330,017.00	77,998.47	150,838.30	519,753.80	66,825.25	119,220.40	354,467.90
2014	74,772.49	138,535.60	374,155.20	84,364.18	165,504.10	585,829.90	73,637.94	136,396.90	438,116.60
2015	81,185.61	153,785.60	390,981.30	95,943.81	194,165.70	728,564.00	81,192.43	154,382.10	522,820.90

Note: This table compares the value of sales, in millions of COP pesos, observed empirically in a sample of 1,772 Colombian firms versus the theoretically expected under lognormal and log-SNP distributions. The values 10%, 5% and 1% are percentiles of the distributions.

**Fig 3:**
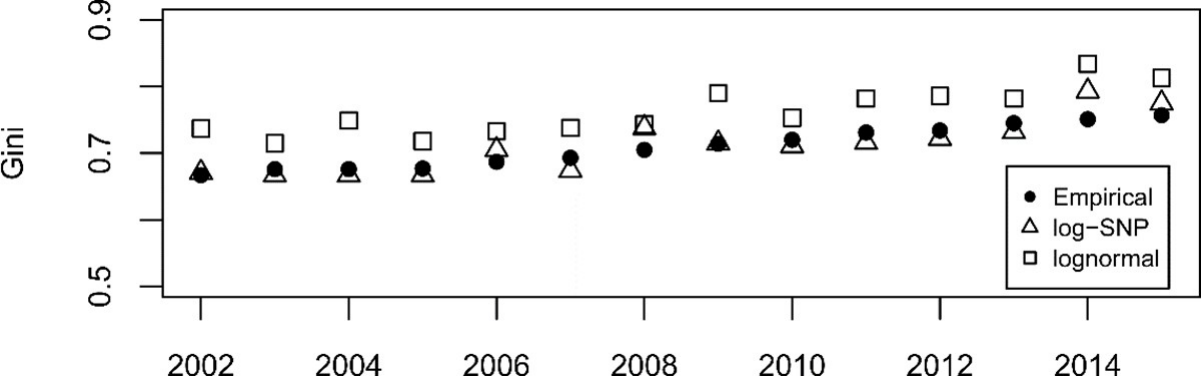
Dynamics of the empirical and theoretical value of the Gini index. The figure compares the time evolution of the empirical values of the Gini index (solid circle) and the theoretically estimated values under a lognormal (square) and log-SNP (triangle) specification.

Eq (5) was employed to measure the Gini index for the level of sales of each company in the sample. The dynamics of the values of this index measured using empirical data and data adjusted theoretically for both distributions is shown in Fig 3. The lognormal distribution tended to overestimate the level of economic concentration, which is consistent with the results presented in Table 3. Moreover, these results are reinforced by those of the KS test for the empirical Gini index and each distribution. For the lognormal distribution (log-SNP), the p-value was 0.002 (0.987) using the KS test, indicating that this distribution was not adequate (null hypothesis could not be rejected) at the usual confidence levels. The existing gap between the extreme values of the FS distribution is relevant in the measurement of economic concentration. When the tail values significantly affect the concentration measure, their inclusion or exclusion is not trivial [6].

These results may be caused by the large proportion of SMEs in Colombia. When measuring the distribution of FS for the total economy, the lower tail is significant. Note that an advantage of the log-SNP distribution is that it allows to measure both tails more adequately. Although in Colombia, as in other economies, small firms tend to grow faster than large firms, the latter are more likely to advance in the upper percentile. In the case of SMEs, owners are often in financial distress and face many obstacles that hinder the growth of their firms. One of them is the possibility of accessing credit to undertake investment projects and reach larger sizes. According to a study conducted by Galindo and Micco [52], most SMEs report difficulties in obtaining financing, which is mainly intense in periods of uncertainty. For small firms, the existence of information asymmetries aggravates the problem and the difficulties in raising funds (although the available information on their financial situation is scarce and poor quality). In addition, many young firms lack credit history and, in most cases, collateral to provide as a guarantee.

**Fig 4:**
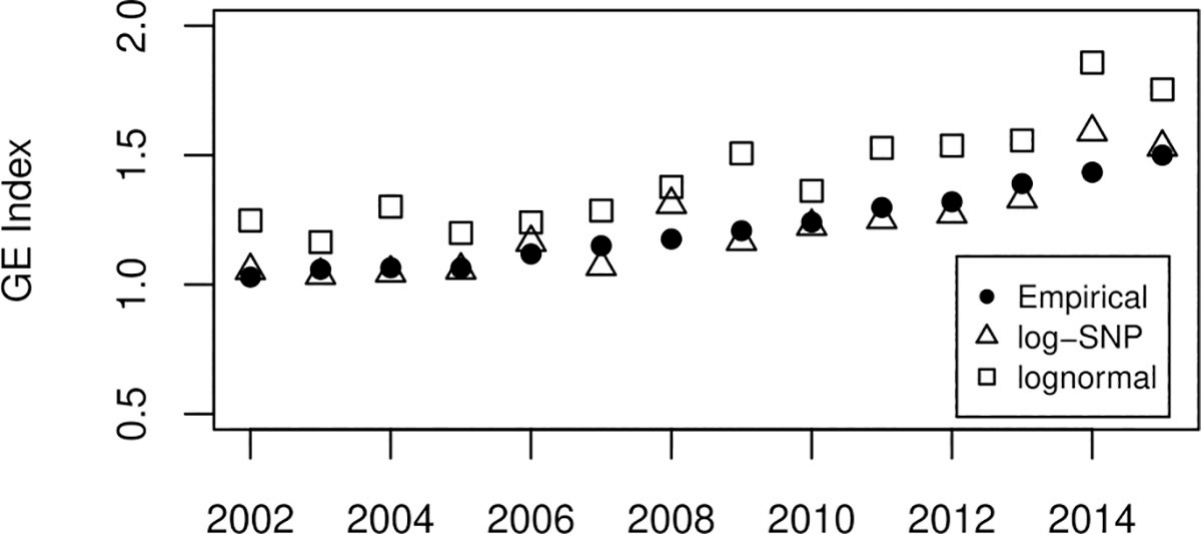
Dynamics of the empirical and theoretical value of the GE index. The figure compares the time evolution of the empirical values of the GE index with alpha = 0 (solid circle) and the theoretically estimated values under a lognormal (square) and log-SNP (triangle) specification.

To validate the robustness of the economic concentration measure obtained from the Gini index, we use the Generalized Entropy (GE) index as it is a more sensitive measure to changes in tails [92]. Specifically, we use the Mean Logarithmic Deviation (MLD), which corresponds to the GE with *alpha* = 0. This concentration measure is more sensitive to upper tail values. As shown in Fig 4, the evolution of the concentration measure is similar to that obtained with the Gini index.

### 6.3. Determinants of firms’ growth and the evidence on Gibrat’s law

The results of the system GMM estimator for three dynamic panel models and the statistical tests for analyzing the estimations provided by the models are shown in Table 4. First, the validity of the instruments was assessed using the Hansen test. This test allowed the detection of the overidentification of the model when the heteroscedastic weight matrix was used in the estimation and, therefore, it was appropriate for analyzing the two-step estimates of the table. In the three estimated models, all explanatory variables were considered endogenous (except for age) and were instrumented. The results supported the validity of the instruments used.

**Table 4:** Determinants of business growth.

	Model 1	Model 2	Model 3
Growth_i,t-1_	-0.1988***	-0.1795***	-0.1802***
	(0.000)	(0.000)	(0.000)
Growth_i,t-2_	-0.0837***	-0.0737***	-0.0740***
	(0.000)	(0.000)	(0.000)
log(Sales_i,t-1_)	-0.0640***	-0.1372***	-0.1347***
	(0.002)	(0.002)	(0.001)
logAge_it_		32.2789**	30.6995**
		(0.059)	(0.061)
[logAge_it_]^2^		-4.9241**	-4.6812**
		(0.061)	(0.064)
Leverage_i,t-1_		1.6136***	1.5566***
		(0.009)	(0.008)
ROE_i,t-1_			-0.0004
			(0.571)
Constant	-1.0254***	50.7655**	-48.2463**
	(0.001)	(0.062)	(0.064)
Observations	24808	24808	24808
Firms	1172	1172	1172
Instruments	7	9	11
Wald Test	148.12	114.54	115.24
	(0.000)	(0.000)	(0.000)
Hansen Test	2.27	0.49	0.78
	(0.518)	(0.782)	(0.854)
Arellano-Bond Test for AR(1)	-14.88	-6.17	-6.66
	(0.000)	(0.000)	(0.000)
Arellano-Bond Test for AR(2)	-1.48	1.28	1.21
	(0.138)	(0.199)	(0.226)

Note: Models 1, 2 and 3 correspond to different estimates of firm growth carried out using the System-GMM estimator. The sample is composed of 1,772 Colombian firms in all sectors of the economy. The data is collected over a 14-year period from 2002 to 2015. Growth is the firm’s growth, calculated as the first difference of the natural logarithm of sales. Sales is the value of net sales in Colombian peso (COP) deflated by Colombian Consumer Price Index (CPI) using 2008 price base. Age corresponds to the age of the company measured in years. Leverage is calculated as the sum of long-term debt and short-term debt divided by total assets. ROE is calculated as net income divided by equity.

*, **, *** indicate levels of significance at 10%, 5% and 1%, respectively. P-values in parentheses.

Second, to achieve consistent estimation of the system GMM, which uses lagged differences or levels as instruments, correlation analysis of the residuals is performed by the Arellano and Bond test. A first-order serial correlation was expected in these models because the residuals in the first differences should be correlated by construction. However, the validity of these models was confirmed only in cases in which a second-order serial correlation was not found. This condition was met by adding a second lag of the endogenous dependent variable in the models [51].

The three estimated models use FG as the dependent variable. Model 1 included the lagged growth and FS as explanatory variables, and Model 2 included age and leverage, and Model 3 included profitability. The lagged growth variables were significant, confirming the dynamic nature and the persistence of FG, which provided evidence against Gibrat’s law and confirmed *Hypothesis 1* [5, 51, 64, 72, 75]. The results show a negative impact of past growth on the contemporary one. This result may be because, in Colombia, high-growth firms only represent about 5% of the total number of firms, like what is found in other countries worldwide. Firms do not grow at more than double digits, and in the case of getting a positive coefficient would imply higher growth year after year. To have sustained growth, firms should keep high levels of investment, especially in R&D, and increased productivity levels [50].

Similarly, all three models showed evidence of a correlation between FG and FS. The estimated coefficient was negative and significant, corroborating *Hypothesis 2*. Small businesses seek high growth rates to achieve a minimum efficient size [8, 68, 71]. Most Colombian firms are in the SMEs segment. This phenomenon is likely explained by the fact that new businesses start with a small size and then increase their size conditioned to their ability to survive. Therefore, small companies must overgrow to survive. Hence, it is relevant that government creates policies to accompany companies in their early stages.

Models 2 and 3 provided evidence on the effect of firm age on growth. There was a positive and significant (p<0.1) linear relationship between these two variables, confirming *Hypothesis 3a*. This result is like that reported by Das [49] and Shanmugam and Bhaduri [81] for a developing economy. Furthermore, the effect of age in its quadratic form indicates that FG is lower as the surviving companies age, which corroborates *Hypothesis 3b*. In Colombia, the proportion of firms with more than ten years within the large segment is around 70%, while for the small segment, it is 21% [50]. As firm get older, they acquire a larger size and a more significant proportion within the large segment. This fact leads that these companies find it easier to access credit and thus expand their production capacity and invest in R&D.

On the other hand, studies have shown that Latin American companies exhibit higher-than-expected leverage because economic concentration is significantly higher than that in developed countries [36, 37]. In this respect, leverage plays an essential role as a determinant of FG. Models 2 and 3 indicated that this variable had a positive and significant coefficient as reported by Huynh and Petrunia [51] and Barba Navaretti et al. [8], and this result confirms *Hypothesis 4*. Colombian firms face credit constraints that diminish as they mature and grow. Historically, small Colombian firms have had less access to credit than large companies [52]. However, credit remains the principal source of financing since access to the capital market is limited. For example, the Colombian Stock Exchange (BVC, in its Spanish acronym) requires, among other things, that firms have at least 100 shareholders at the time of issuing new shares. These requirements are very limiting due to the high concentration of ownership observed in Colombian and Latin American firms.

Furthermore, Model 3 proposes the analysis of profitability as a determinant of growth. As a result, a negative and statistically non-significant coefficient was obtained, with which we cannot provide conclusions about *Hypothesis 5*. However, this result may provide evidence of non-compliance with the pecking order theory in Latin American companies, as observed in previous studies in the region [37]. This result is in line with *Hypothesis 4*.

## 7. Conclusions

This paper sheds light on the compliance of Gibrat’s law using a sample of 1,772 Colombian companies collected between 2002 and 2015 and comparing the performance of FS distribution using the lognormal distribution [1] and log-SNP distribution [31]. The latter distribution nests the lognormal distribution and includes new parameters that can better assess the characteristics of the upper and lower quantiles corresponding to larger and smaller companies. The results indicate that the lognormal distribution tends to systematically overestimate the expected values in the far end of the distribution tails but the log-SNP becomes a flexible method to fit them more accurately.

This finding emphasizes the need to propose other methodologies to obtain more reliable information on the level of economic concentration. In this line, we demonstrate analytically that the Gini index has a better result if it is fitted with SNP methods formulated in terms of the log-SNP distribution. In fact, the lognormal distribution tends to overestimate the level of economic concentration. This is because the log-SNP distribution is more flexible than the lognormal distribution when the data are skewed, and there are possible jumps in the tails due to outliers.

Furthermore, to test the validity of Gibrat’s law and investigate on the determinants of FG, we estimated the relationship between this variable and FS, as well as other potentially explanatory variables: age, leverage, and profitability. Based on the system GMM estimator proposed by Blundell and Bond [55], we conclude that Gibrat’s law does not apply to the selected sample in Colombia. The FG rates strongly depended on the FS and presents a significant persistence over time. We also find that some company characteristics were fundamental determinants of FG, particularly firm age and leverage had a significant impact on growth. There was no evidence of a positive correlation between profits and FG, which can be explained by the high level of economic concentration in Latin American firms and by their focus on leverage.

These results represent a valuable contribution, not only for researchers on Industrial Organization, but also for policymakers, since the knowledge about FS distribution and their determinants of growth, help to forecast industrial concentration and its impact on economic cycles and, consequently, implement adequate antitrust and economic policies. Policymakers should concentrate their efforts on promoting high-growth firms during their early stages to reach a size that will allow them to survive. It is noteworthy that, especially in the early stages, the business scale is small and manageable, giving these firms the ability to adapt to market niches that large firms do not necessarily focus on. Without government support, these firms face more significant difficulties in growing organically, which would cause the concentration in large firms to increase. Therefore, government should create policies and incentives that enable these companies to expand their production capacity, increasing their likelihood of accessing credit, and increase their productivity.

However, there are still various unsolved problems that should be considered in future research, e.g. addressing some limitations of the data coming from Latin American institutions and the extension of the analysis at the sectoral level. The degree of heterogeneity of the results for different sectors could provide a richer economic structure that could be hidden by the aggregated analysis. In addition to this, Gibrat’s law can be tested considering all the companies within an industry or a specific economy and time interval, including the companies that did not survive.
